# Combined Off-Pump Coronary Artery Bypass Grafting and Lung Resection in Patients with Lung Cancer Accompanied by Coronary Artery Disease

**DOI:** 10.21470/1678-9741-2018-0126

**Published:** 2018

**Authors:** Ali Yeginsu, Mustafa Vayvada, Burcin C. Karademir, Atakan Erkılınç, Ahmet Erdal Tasci, Fuat Buyukbayrak, Emre Gurcu, Cemal Asım Kutlu

**Affiliations:** 1 Department of Thoracic Surgery, Kartal Kosuyolu Yuksek Ihtisas Training and Research Hospital, Kartal, Istanbul, Turkey.; 2 Department of Cardiovascular Surgery, Kartal Kosuyolu Yuksek Ihtisas Training and Research Hospital, Istanbul, Turkey.; 3 Department of Anesthesia and Reanimation, Kartal Kosuyolu Yuksek Ihtisas Training and Research Hospital, Istanbul, Turkey.; 4 Department of Cardiovascular Surgery, Okan University School of Medicine, Istanbul, Turkey.; 5 Kartal Kosuyolu Yuksek Ihtisas Training and Research Hospital, Department of Anesthesia and Reanimation, Istanbul, Turkey.; 6 Yuzuncu Yil University School of Medicine Gaziosmanpasa Hospital, Department of Thoracic Surgery, Istanbul, Turkey.

**Keywords:** Lung Neoplasms, Coronary Artery Disease, Coronary Artery Bypass, Off-Pump

## Abstract

**Introduction:**

Optimal surgical approach for the treatment of resectable lung cancer accompanied by coronary artery disease (CAD) remains a contentious issue. In this study, we present our cases that were operated simultaneously for concurrent lung cancer and CAD.

**Methods:**

Simultaneous off-pump coronary artery bypass surgery (OPCABG) and lung resection were performed on 10 patients in our clinic due to lung cancer accompanied by CAD. Demographic features of patients, operation data and postoperative results were evaluated retrospectively.

**Results:**

Mean patient age was 63.3 years (range 55-74). All patients were male. Six cases of squamous cell carcinoma, three of adenocarcinoma and one case of large cell carcinoma were diagnosed. Six patients had single-vessel CAD and 4 had two-vessel CAD. Three patients underwent OPCABG at first and then lung resection. The types of resections were one right pneumonectomy, three right upper lobectomies, one right lower lobectomy, three left upper lobectomies, and two left lower lobectomies. Reoperation was performed in one patient due to hemorrhage. One patient developed intraoperative contralateral tension pneumothorax. One patient died due to acute respiratory distress syndrome at the early postoperative period.

**Conclusion:**

Simultaneous surgery is a safe and reliable option in the treatment of selected patients with concurrent CAD and operable lung cancer.

**Table:** 

Abbreviations, acronyms & symbols				
ACT	= Activated clotting time		LC	= Lung cancer
ARDS	= Acute respiratory distress syndrome		LND	= Lymph node dissection
CABG	= Coronary artery bypass grafting		LUL	= Left upper lobe
CAD	= Coronary artery disease		MRI	= Magnetic resonance imaging
CPB	= Cardiopulmonary bypass		OPCABG	= Off-pump coronary artery bypass grafting
DAPT	= Dual antiplatelet therapy		PCI	= Percutaneous coronary intervention
ECG	= Electrocardiogram		PET/CT	= Positron emission tomography
IMA	= Internal mammary artery		RUL	= Right upper lobe

## INTRODUCTION

As the elderly population increases, there are an increasing number of patients who are diagnosed with lung cancer (LC) and have concurrent heart disease requiring surgical intervention. According to The Society of Thoracic Surgeons database, 20.9% of the patients undergoing pulmonary resection for LC have coronary artery disease (CAD); however, only 0.4-0.5% of these patients required coronary artery bypass grafting (CABG)^[1-3]^.

The choice of the ideal surgical procedure is still controversial. Percutaneous coronary intervention (PCI), followed by lung resection, required months of delay in tumor management due to the use of dual antiplatelet therapy (DAPT) and stent thrombosis after cessation of DAPT is the main problem. In staged operations, the cardiac procedure was performed first and after a recovery period of 2 to 8 weeks, pulmonary resection was performed^[3]^. Disadvantages of the staged approach are complications arising from the consequent administration of anesthesia, risk of disease progression due to delayed treatment, patients' anxiety, cost of treatment, hospital stay^[3,4]^. On the other hand, simultaneous procedures have a longer operation time and lead to increased risks of operative bleeding, tissue edema and immunosuppression due to cardiopulmonary bypass (CPB)^[3-6]^.

Off-pump coronary artery bypass grafting (OPCABG) technique has been gaining popularity since the last decade, because it does not require CPB. Recently, several papers showed that simultaneous OPCABG and lung resections may be safe and feasible in combined CAD and LC^[3,5-8]^. We presented our patients who underwent simultaneous surgery for concurrent CAD and LC in our clinic.

## METHODS

We retrospectively analyzed patients submitted to simultaneous OPCABG and lung resection from 2014 to 2018. All patients evaluated preoperatively. Respiratory, cardiovascular and other systemic assessments were meticulously performed. All LCs were biopsy-proven and metastatic state was eliminated by positron emission tomography (PET/CT) and cranial magnetic resonance imaging (MRI). If no suspicious mediastinal lymph node was detected on chest CT and PET-CT examinations, preoperative invasive staging was not applied. CAD was evaluated with angiography. All patients were discussed in our multidisciplinary council before the operation.

All patients were operated on electively. CABG was performed at first in 3 patients and then lung resections. Median sternotomy approach was used in all patients. A left anterior thoracotomy was accompanied in one. Patients were heparinized (100-200 U/kg) prior to surgery to adjust the activated clotting time (ACT) between 200-400 seconds. The pericardium was opened. In order to slightly twist the heart counterclockwise and lift it to provide more comfort during the revascularization procedure, deep suspending sutures were placed to the pericardium, taking care not to interfere with the hemodynamic stability. Both the saphenous vein and the left internal mammary artery were prepared as grafts. By suturing the proximal and distal portions of the coronary artery that were anastomosed with 4-0 Prolene, antegrade and retrograde blood flow was blocked. If there were ST and T changes in electrocardiogram (ECG) during the suspension, intracoronary shunt was applied. In order to ensure a stationary anastomosis site, the Octopus III coronary stabilizer was used. After achieving controlled bradycardia via intravenous administration of selective beta-1 blocker (metoprolol tartrate), distal anastomoses were applied using 7-0 Prolene suture. In order to provide a blood-free anastomosis site, a sterile air blower was used. After placing a side clamp in the ascending aorta, proximal anastomoses were made with 5-0 Prolene suture. Following hemostasis, heparin neutralization was provided with protamine at a 1:1.3 ratio.

Selective lymph node dissection (LND) was performed in all patients^[9]^. Stations #2R and #4R were dissected for right upper lobe (RUL) tumor and stations #4, #5 and #6 were dissected for left upper lobe (LUL) tumors. Stations #7, #8 and #9 were dissected for lower lobes in both sides. If the intraoperative frozen section study was positive, then systematic LND was performed. Histopathological examination was performed on all surgical resection materials. LC staging was made according to international TNM staging system (primary tumor spread; regional lymph node involvement; intrathoracic or distant metastasis).

After discharge, follow-up visits were scheduled at 3, 6, 12, 18 and 24 months and then every year. All patients were referred to an oncological council after discharge.

## RESULTS

All patients were male, and the mean patient age was 63.3 years (range 55-74). The main patient complaints were difficulty in breathing, chest pain and palpitations. Six cases of squamous cell carcinoma, three of adenocarcinoma and one case of large cell carcinoma were diagnosed. Six patients had single-vessel CAD and four had two-vessel CAD. Seven patients were in stage 1, two in stage 2 and one in stage 3. As comorbidity factors, four patients had diabetes mellitus, four hypertension and one had chronic obstructive pulmonary disease (Table 1).

**Table 1:** Patient demographics.

Patient	Age	Gender	BMI	Tumor cell type	TNM	Stage	Comorbidity
1	62	M	24	Squamous cell carcinoma	T1aN0M0	Ia	HT, COPD
2	63	M	26	Adenocarcinoma	T2aN0M0	Ib	DM
3	58	M	21	Adenocarcinoma	T2bN0M0	IIa	HT
4	62	M	24	Large cell carcinoma	T3N0M0	IIb	-----
5	55	M	28	Squamous cell carcinoma	T1aN0M0	Ia	DM
6	74	M	26	Squamous cell carcinoma	T1bN0M0	Ia	DM
7	62	M	22	Squamous cell carcinoma	T2aN2M0	IIIa	DM, HT
8	72	M	19	Squamous cell carcinoma	T3N1M0	IIIa	HT
9	68	M	28	Squamous cell carcinoma	T2aN0M0	1b	-----
10	57	M	21	Adenocarcinoma	T1aN0M0	Ia	-----

BMI=body mass index; COPD=chronic obstructive pulmonary disease; DM=diabetes mellitus; HT=hypertension; M=male

The mean operation time was 292 (range 241-336) minutes. Median sternotomy was performed in all cases. An additional anterior thoracotomy was needed in one patient submitted to left lower lobectomy. Coronary revascularization was performed first in eight of the 10 cases. Left internal mammary artery (IMA) graft was used in six patients and saphenous vein graft was used in four patients (Tables 2 and 3). The mean number of red blood cell suspension transfusions was 1.6 (range 0-4). None of the patients required cardiopulmonary support. Seven patients required inotropic agents.

**Table 2:** Operative data of the patients.

Patient	Type of lung resection	CABG vessel	Graft	Incision	Lung resection	Red blood cells suspension (Unit)
1	LUL	Single	SVG	Sternotomy	After OPCABG	3
2	LUL + RULWR	Two	Left IMA	Sternotomy	After OPCABG	2
3	LLL + RULWR	Single	SVG	Sternotomy + Left anterior thoracotomy	Before OPCABG	4
4	LLL	Single	SVG	Sternotomy	Before OPCABG	0
5	RUL	Single	SVG	Sternotomy	After OPCABG	1
6	RLL	Single	Left IMA	Sternotomy	After OPCABG	0
7	RUL	Two	Left IMA	Sternotomy	After OPCABG	1
8	RP	Two	Left IMA	Sternotomy	After OPCABG	3
9	RUL	Single	Left IMA	Sternotomy	After OPCAB	2
10	LUL	Two	Left IMA	Sternotomy	After OPCAB	0

CABG=coronary artery bypass grafting; IMA=internal mammary artery; LLL=left lower lobectomy; LUL=left upper lobectomy; OPCABG=Off-pump coronary artery bypass grafting; RLL=right lower lobectomy; RP=right pneumonectomy; RUL=left upper lobectomy; RULWR=right upper lobe wedge resection; SVG=saphenous vein graft

**Table 3:** Revascularization characteristics of the patients.

Patient	CABG vessel	Location	Graft	Anastomosis technique
1	Single	LAD	SVG	End-to-side
2	Two	LAD and D1	LIMA	Sequential technique (D1 – Side-to-side) (LAD – End-to-side)
3	Single	LAD	SVG	End-to-side
4	Single	LAD	SVG	End-to-side
5	Single	OM1	SVG	End-to-side
6	Single	LAD	LIMA	End-to-side
7	Two	LAD and D1	LIMA	Sequential technique (D1 – Side-to-side) (LAD – End-to-side)
8	Two	LAD and D1	LIMA	Sequential technique (D1 – Side-to-side) (LAD – End-to-side)
9	Single	LAD	LIMA	End-to-side
10	Two	LAD and D1	LIMA	Sequential technique (D1 – Side-to-side) (LAD – End-to-side)

CABG=coronary artery bypass grafting; LAD=left anterior descending artery; D1=first diagonal arterial branch of LAD; LIMA=left internal mammary artery; SVG=saphenous vein graft; OM1=obtuse marginal artery

One right pneumonectomy, three right upper lobectomies, one right lower lobectomy, three left upper lobectomies, and two left lower lobectomies were performed. Additional RUL wedge resections were made for suspicious nodules in the contralateral lung in two patients. Intraoperative frozen section examination indicated benign lesions. No N2 disease was detected by selective LND intraoperatively.

The mean intensive care length of stay was 3.9 days (range 4-7). The mean hospital length of stay was 14.4 days (range 5-19). None of the patients required CPB. We saw four major complications in patients. Bleeding requiring reoperation was seen in one patient. Another patient had perioperative tension pneumothorax. We opened the contralateral pleura and introduced a chest tube at the time of closure. One patient suffered from atrial fibrillation on the second day and recovered with antiarrhythmic medication. The last patient presented acute respiratory distress syndrome (ARDS) at the early postoperative period and died due to multiple organ failure 5 days after the operation. Perioperative or postoperative myocardial infarction was not seen. The mean duration of postoperative follow-up was 19.1 months (range 6-35). One patient died in the 35^th^ month of follow-up of tumor dissemination (Table 4).

**Table 4:** Postoperative results.

Patient	ICU stay (days)	Hospital stay (days)	Complication	Mortality	Survival
1	2	16	-----	Dead	35 months
2	5	5	ARDS, multiorgan failure	Dead	5 day
3	2	19	-----	Alive	30 months
4	4	18	-----	Alive	6 months
5	7	13	Reoperation for bleeding	Alive	28 months
6	4	17	-----	Alive	27 months
7	4	11	-----	Alive	25 months
8	5	12	Perioperative tension pneumothorax	Alive	20 months
9	2	14	-----	Alive	6 months
10	4	19	Atrial fibrillation	Alive	9 months

ARDS=adult respiratory distress syndrome; ICU=intensive care unit

## DISCUSSION

In combined CAD and LC, treatment options are: 1) PCI followed by lung resection; 2) staged CABG and lung resection; 3) simultaneous CABG and lung resection^[3]^. CABG can be performed with or without CPB. Regarding the treatment of these patients, each technique has its own advantages and disadvantages.

Balloon angioplasty with stent placement is a safe and effective method, but it has not been recommended for patients with left main CAD and with three-vessel disease. It has increased risk of operative bleeding related to the use of antithrombotic medication^[7,10,11]^. Timing of cancer surgery is debatable due to stent thrombosis after cessation of DAPT. At least, 3 month delay between PCI and lung resection has been recommended^[6,12]^. Fernandez et al.^[13]^ showed increased perioperative thrombosis or myocardial infarction and mortality in patients with stents compared to non-stent patients. Stent placement in the 12 months prior to surgery significantly increased 30-day cardiac events (9.3%) and mortality (7.7%).

The use of CPB in CABG is still a great dilemma due to its side effects such as: increased surgical bleeding, triggering the systemic inflammatory response syndrome; increased capillary bed permeability; and pulmonary edema, leading to impairment in immune system^[3-6]^. The purpose of the OPCABG is to protect physiological systems from adverse effects resulting from the use of CPB and related methods. Performing myocardial revascularization while the heart is still beating helps to reduce postoperative complications and immunosuppression^[14]^. Avoidance of CPB results in reduced pulmonary function disturbances, decreased incidence of postoperative atrial fibrillation, and reduced need for blood transfusion, perioperative intra-aortic balloon pump and possible tumor cell dissemination^[15,16]^.

Patients undergoing simultaneous surgery with CPB present complications and mortality rates significantly higher than those expected in staged procedures^[3,5]^. Bleeding rate is between 0-16%^[3]^ and the infectious complications are about 14%^[17]^ in CPB used in simultaneous surgery. Operative mortality, 1- and 5-year survival were 0-20.8%, 79-100% and 34.9-85%, respectively, in patients undergoing simultaneous surgery, while 0-10%, 72.7% and 53%, respectively, in patients undergoing staged surgery^[3,5]^. On the other hand, OPCABG has been shown to be a safe and reliable method for simultaneous surgery^[3,5-8]^(Table 5). Bleeding risk and mortality ranged from 0 to 8% and 0 to 6.6%, respectively. Five-year survival was 13 to 68%^[3,5]^. In our patients, bleeding rate and operative mortality occur in one (10%) patient.

**Table 5:** Outcomes of simultaneous OPCABG and lung tumor resection in the literature.

Author, study	Outcomes	Conclusion
• Saxena et al.^[8]^ • Cohort study (2004) • Six patients underwent OPCABG and LR between 2000-2004 • Mean age was 67.6 years (58-75) • Single- to 3-vessel CAD and stage 1-2 tumors	• Patients were followed up for 9 months to 3 years • Prolonged airleak and pleural space in 2 patients • No CAD recurrence • No early mortality • Late mortality: 2/6 patients (33%) • Mean hospital stay: 12.5 days	• Authors concluded that combined surgery is a safe approach in patients with concomitant CAD and lung cancer
• Schoenmakers et al.^[6]^ • Cohort study (2007) • 28 patients underwent CABG and 15 underwent OPCABG and LR between 1994-2005 • Mean age was 66 years in CABG group and 71 years in OPCABG group • Single- to 3-vessel CAD and stage 1-3 tumors	• Hospital mortality was 2% in CABG and 1% in OPCABG group (*P*<0.05). • Reexploration, cardiac and pulmonary complications. • There were more pulmonary complications in the CABG group ( *P*=0.04). • Mean survival was 5.25 years in CABG group and 3 years in OPCABG group (*P*=0.09) • Two- and 5-year survival rates are 64% and 46% in CABG group and 47% and 13% in OPCABG group ( *P*<0.01). • Mean hospital stay was 14 days in CABG group and 12 days in OPCABG group	• Authors believe that the results of their study did not show evidence that OPCABG is a superior treatment to CABG in patients with combined cardiac and lung pathology • On the other hand, OPCABG group has fewer and older patients. And they have more advanced lung cancer, therefore more studies were needed for evaluation
• Dyszkiewicz et al.^[2]^ • Cohort study (2008) • 25 patients underwent combined OPCABG and LR from 2001 to 2006 • Mean age was 63 years (57-75) • Majority of patients have 2-vessel CAD and stage 1-3 tumors • 13 patients had undergone PCI and/or stent implantation before lung cancer was diagnosed	• Patients were followed up for 8 months to 5 years • No perioperative death and new MI • Rethoracotomy in 1 patient, atrial fibrillation in 6, atelectasis in 3, prolonged airleak in 3, bronchopleural fistula in 1 • 3-year survival was 50% • Recurrence of cancer was 55% and was the only statistically significant factor with an impact on survival (*P*<0.01) • Mean hospital stay was 8 days (6-28)	• Combined surgery is a safe and effective treatment in patients with concomitant unstable CAD and lung cancer • According to the author, combined surgery requires different surgical skills
• Ma et al.^[7]^ • Cohort study (2016) • 34 patient underwent combined OPCABG and LR from 2003 to 2014 • Mean age was 65 years (54-77) First OPCABG was performed and then LR • Single- to 3-vessel CAD and stage 1-3 lung cancer patients	• Patients were followed up for 5 to 60 months • No perioperative death • No perioperative MI • Two patients had lung infection, 3 had atelectasis and 5 had arrhythmias • Three- and 5-year mortality were 75 and 67%, respectively • Recurrence occured in 7 patients • Mean hospital stay was 17±6 days	• Combined OPCABG and LR is a safe and effective treatment option with good long-term survival rates • Additionally, this procedure is a safe option in patients with 3-vessel CAD who are not candidates for PCI
• Yeginsu et al. (present article) • Cohort study (current) • 10 patients underwent combined OPCABG and LR from 2014 to 2018 • Mean age was 63 (55-74) years •Single to 2 vessel CAD and stage 1-2 lung cancer patients	• Patients were followed up for 6 to 35 months • No operative death • No operative MI • One patient (10%) died in early and 1 (10%) died in the late postoperative period • Major complications were bleeding, ARDS, tension pneumothorax and atrial fibrillation • Mean hospital stay was 14.4 days (5-19)	• Combined OPCABG and LR is a safe and reliable surgical method; however, data is not sufficient to conclude that it is the best approach for the patient with concomitant CAD and lung cancer

ARDS=adult respiratory distress syndrome; CABG=coronary artery bypass grafting; CAD=coronary artery disease; OPCABG=off-pump coronary artery bypass grafting; LR=lung resection; MI=myocardial infarction; PCI=percutaneous coronary intervention

In technical aspects of simultaneous operations, choice of procedural approach, timing and sequence of surgical steps, and adequacy of procedure for tumor surgery are important issues.

Median sternotomy should be the standard approach for simultaneous heart and lung operations^[2-5,8]^. Compared to posterolateral thoracotomy, pulmonary dysfunction and postoperative pain are lower with median sternotomy. However, sternotomy may be inefficient to provide an adequate field of vision for lung resections. The process of dissection and traction of the heart may lead to arrhythmias and hemodynamic instability, especially for tumors localized to left lower lobe. By opening the right pleura and pericardium and pushing the heart to the right side, a better field of vision may be accomplished. Single-lung ventilation, complete mobilization of the lung and myocardial revascularization in the first place may facilitate this maneuver^[18,19]^. An additional left anterior thoracotomy and/or thoracoscopic assistance are very helpful for an adequate exposure. We performed sternotomy plus anterior thoracotomy in one patient with left lower lobe tumor and we found no difficulty during resection.

The main goal of a simultaneous operation in patients with lung tumor and CAD should be to avoid myocardial ischemia during lung resection. Therefore, OPCABG should be performed first rationally^[7]^. However, it is recommended that lung resection should be performed first when CPB is used^[5]^. In addition, some technical difficulties, such as tumor size and location, dense pleural adhesions, use of both IMAs, may require lung resection first. We performed lung resection first in two patients because the tumors located centrally in left lower lobe and there were dense pleural adhesions and we concerned about injuring the grafts when we resected the lobes.

Another important aspect in LC surgery with median sternotomy is the adequate dissection of mediastinal and hilar lymph nodes. Single-lung ventilation, dividing inferior pulmonary ligament and intrapleural liberation, may help to achieve an adequate field of vision for LND. With sternotomy, hilar, interlobar, lobar, segmental and inferior pulmonary lymph nodes at both mediastinal, and subcarinal lymph nodes on the left side may be sampled^[8]^. Although systematic LND is recommended^[20]^, selective LND is also an acceptable option in LC surgery for adequate tumor cleaning and staging for stage 1 and 2 tumors^[9]^. The reported possible advantage of selective LND is reducing the hospital stay, but its reliability and applicability are still unclear. Multi-institutional randomized clinical trials are needed to answer the question of which is the best option.

## CONCLUSION

In conclusion, simultaneous lung resections and OPCABG in patients with CAD accompanying LC is a safe and reliable surgical method. Several authors reported that it presented less morbidity and mortality compared to other options using CPB. Long-term survival is comparable in the others. On the other hand, data are not sufficient to conclude that combined OPCABG and lung resection are the best approach for the patient with concomitant LC and CAD. We need larger randomized studies for certainty.

**Table:** 

Authors' roles & responsibilities	
AY	Substantial contributions to the conception or design of the work; or the acquisition, analysis, or interpretation of data for the work; drafting the work or revising it critically for important intellectual content; final approval of the version to be published; final approval of the version to be published
MV	Drafting the work or revising it critically for important intellectual content; final approval of the version to be published; final approval of the version to be published
BCK	Substantial contributions to the conception or design of the work; or the acquisition, analysis, or interpretation of data for the work; final approval of the version to be published
AE	Final approval of the version to be published
AET	Agreement to be accountable for all aspects of the work in ensuring that questions related to the accuracy or integrity of any part of the work are appropriately investigated and resolved; final approval of the version to be published
FB	Final approval of the version to be published
EG	Drafting the work or revising it critically for important intellectual content; final approval of the version to be published
CAK	Substantial contributions to the conception or design of the work; or the acquisition, analysis, or interpretation of data for the work; final approval of the version to be published
